# Prolonged distal motor latency of median nerve does not improve diagnostic accuracy for CIDP

**DOI:** 10.1007/s00415-021-10672-w

**Published:** 2021-06-26

**Authors:** Emanuele Spina, Pietro Emiliano Doneddu, Giuseppe Liberatore, Dario Cocito, Raffaella Fazio, Chiara Briani, Massimiliano Filosto, Luana Benedetti, Giovanni Antonini, Giuseppe Cosentino, Stefano Jann, Anna Mazzeo, Andrea Cortese, Girolama Alessandra Marfia, Angelo Maurizio Clerici, Gabriele Siciliano, Marinella Carpo, Marco Luigetti, Giuseppe Lauria, Tiziana Rosso, Guido Cavaletti, Erdita Peci, Stefano Tronci, Marta Ruiz, Stefano Cotti Piccinelli, Angelo Schenone, Luca Leonardi, Luca Gentile, Laura Piccolo, Giorgia Mataluni, Lucio Santoro, Eduardo Nobile-Orazio, Fiore Manganelli, The Italian CIDP Database Study Group, Pietro Emiliano Doneddu, Giuseppe Liberatore, Francesca Gallia, Eduardo Nobile-Orazio, Rozzano, Erdita Peci, Dario Cocito, Stefano Tronci, Raffaella Fazio, San Raffaele, Fiore Manganelli, Lucio Santoro, Emanuele Spina, Enrica Pisano, Marta Ruiz, Chiara Briani, Stefano Cotti Piccinelli, Massimiliano Filosto, Alessandro Beronio, Luana Benedetti, Antonio Toscano, Luca Gentile, Anna Mazzeo, Giorgia Mataluni, Girolama Alessandra Marfia, Laura Piccolo, Elisa Vegezzi, Andrea Cortese, Giuseppe Cosentino, Brigida Fierro, Verrengia Elena Pinuccia, Stefano Jann, Elisa Bianchi, Ettore Beghi, Angelo Maurizio Clerici, Federica Scrascia, Marinella Carpo, Martina Garnero, Angelo Schenone, Marco Luigetti, Mario Sabatelli, Patrizia Dacci, Giuseppe Lauria, Luca Leonardi, Giovanni Antonini, Tiziana Rosso, Rika Schirinziand, Gabriele Siciliano, Claudia Balducci, Guido Cavaletti

**Affiliations:** 1 Department of Neuroscience, Reproductive Sciences and Odontostomatology, University of Naples “Federico II”, Via Pansini, 5, 81025 Naples, Italy; 2 Neuromuscular and Neuroimmunology Service, IRCCS Humanitas Clinical and Research Institute, Rozzano, Milan Italy; 3 Presidio Sanitario Major, Istituti Clinici Scientifici Maugeri, Turin, Italy; 4 Division of Neuroscience, Department of Neurology, Institute of Experimental Neurology (INSPE), San Raffaele Scientific Institute, Milan, Italy; 5 Department of Neuroscience, Neurology Unit, University of Padova, Padova, Italy; 6 Center for Neuromuscular Diseases and Neuropathies, Unit of Neurology, ASST ‘Spedali Civili’, University of Brescia, Brescia, Italy; 7 Department of Neuroscience, Rehabilitation, Ophthalmology, Genetics, Maternal and Child Health, University of Genoa and IRCCS San Martino, Genoa, Italy; 8 IRCCS AOU San Martino-IST, Genoa, Italy; 9 Unit of Neuromuscular Diseases, Department of Neurology Mental Health and Sensory Organs (NESMOS), Faculty of Medicine and Psychology, Sant’Andrea Hospital, ‘Sapienza’ University of Rome, Rome, Italy; 10 Department of Brain and Behavioral Sciences, University of Pavia, Pavia, Italy; 11 IRCCS Mondino Foundation, Pavia, Italy; 12 Department of Neuroscience, Niguarda Ca’ Granda Hospital, Milan, Italy; 13 Department of Clinical and Experimental Medicine, Unit of Neurology, University of Messina, Messina, Italy; 14 Molecular Neurosciences, University College London, London, UK; 15 Dysimmune Neuropathies Unit, Department of Systems Medicine, Tor Vergata University of Rome, Rome, Italy; 16 Neurology Unit, Circolo and Macchi Foundation Hospital, Insubria University, DBSV, Varese, Italy; 17 Neurology Unit, Department of Clinical and Experimental Medicine, University of Pisa, Pisa, Italy; 18 Neurology Unit, ASST Bergamo Ovest-Ospedale Treviglio, Treviglio, Italy; 19 UOC Neurologia, Fondazione Policlinico Universitario Agostino Gemelli IRCCS, Rome, Italy; 20 Dipartimento di Neuroscienze, Università Cattolica del Sacro Cuore, Rome, Italy; 21 Unit of Neuroalgology, IRCCS Foundation ‘Carlo Besta’ Neurological Institute, Milan, Italy; 22 Department of Biomedical and Clinical Sciences ‘Luigi Sacco’, University of Milan, Milan, Italy; 23 ULSS2 Marca Trevigiana, UOC Neurologia-Castelfranco Veneto, Treviso, Italy; 24 School of Medicine and Surgery and Experimental Neurology Unit, University of Milano-Bicocca, Monza, Italy; 25 Department of Medical Biotechnology and Translational Medicine, Milan University, Milan, Italy

**Keywords:** CIDP, Neurophysiology, Carpal tunnel syndrome, Diagnostic accuracy, Demyelination

## Abstract

Compression of the median nerve at the carpal tunnel can give demyelinating features and result in distal motor latency (DML) prolongation fulfilling the EFNS/PNS demyelinating criteria for chronic inflammatory demyelinating polyneuropathy (CIDP). Accordingly, being carpal tunnel syndrome (CTS) common in the general population, the EFNS/PNS guidelines recommend excluding the DML of the median nerve when DML prolongation may be consistent with median neuropathy at the wrist from CTS. The main aims of this study were to verify whether the inclusion of DML of the median nerve (when consistent with CTS) could improve electrophysiological diagnostic accuracy for CIDP and if the median nerve at the carpal tunnel was more prone to demyelination. We analyzed electrophysiological data from 499 patients included consecutively into the Italian CIDP Database. According to the EFNS/PNS criteria, 352 patients had a definite, 10 a probable, and 57 a possible diagnosis of CIDP, while 80 were not fulfilling the diagnostic criteria. The inclusion of DML prolongation of median nerve did not improve significantly the diagnostic accuracy for CIDP; overall diagnostic class changed in 6 out of 499 patients (1.2%) and electrodiagnostic class of CIDP changed from not fulfilling to possible in only 2 patients (2.5% of not-fulfilling patients). In conclusion, we can infer that excluding DML prolongation of median nerve does not increase the risk of missing a diagnosis of CIDP thus corroborating the current EFNS/PNS criteria.

## Introduction

Carpal tunnel syndrome (CTS) is the most common nerve entrapment neuropathy worldwide [1]. It is due to compression of the median nerve in the narrow passage from the forearm to the hand, constituted by flexor retinaculum and bones of the wrist. CTS has a prevalence of 50 cases per 1000 people, has female predominance and has an onset in the fifth or sixth decade [1]. The diagnosis of CTS is based on the clinical presentation of relevant symptoms and supported by electrophysiological findings due to the entrapment of the median nerve at the wrist. Distal motor latency (DML) prolongation along with sensory nerve conduction velocity (SNCV) slowing or absence of sensory action potential (SAP) is the hallmark of the electrophysiological diagnosis [2].

Chronic inflammatory demyelinating polyneuropathy (CIDP) is the most common acquired chronic inflammatory neuropathy and its prevalence range from 0.8 to 8.9 cases per 100,000 people.[3]. Diagnosis of CIDP relies on clinical signs and symptoms confirmed by electrophysiological test demonstrating peripheral nerve demyelination, according to the European Federation of Neurological Societies and Peripheral Nerve Society (EFNS/PNS) criteria. Supportive criteria can assist the diagnosis [4, 5].

DML prolongation ≥ 50% above the upper limit of normal (ULN) is a major EFNS/PNS electrophysiological criterion for diagnosing CIDP [4]. However, compression or entrapment itself (e.g. CTS) can give demyelinating features and result in DML prolongation fulfilling EFNS/PNS demyelinating criteria.

Accordingly, the EFNS/PNS guidelines recommend excluding the DML of the median nerve when DML prolongation may be consistent with median neuropathy at the wrist from CTS, that is if DML is associated with SNCV slowing or absence of SAP [4].

Under these premises, the main aim of this study was to verify whether the inclusion of DML of the median nerve (when consistent with CTS) could improve electrophysiological diagnostic accuracy for CIDP.

Moreover, since previous reports on small CIDP samples have not observed more severe demyelination at entrapment sites [6, 7] we decided to verify whether the median nerve at the carpal tunnel was more prone to demyelination in a larger CIDP population.

## Methods

This was a retrospective multicentre cohort study in a large sample of CIDP patients implementing a web database (CINECA, Bologna, Italy) to collect demographical, clinical, and electrophysiological data from patients diagnosed and followed by 22 centers throughout Italy with expertise on CIDP.

The Ethical Committee of each participating Center approved the study and all the patients gave written informed consent. Experienced neurologists with a neuromuscular subspecialty obtained clinical and neurophysiological data. Verification of the diagnostic data for all of the enrolled patients was centralized in the database coordinator center (Humanitas). Data monitoring included diagnosis revision, suspect double entries, missing data, and plausibility checks. Patients with an alternative diagnosis for the neuropathy, an increased titers of anti-myelin-associated glycoprotein IgM antibodies or without available nerve conduction studies were excluded [8].

Based on electrophysiological findings, patients were classified according to EFNS/PNS guidelines in four CIDP diagnostic classes: definite, probable, possible, and not fulfilling. The reasons why patients who did not meet the criteria were equally diagnosed as CIDP was discussed in a recent paper. To note these patients had similar clinical features and frequency of abnormal supportive criteria for the diagnosis of CIDP compared to patients fulfilling EFNS/PNS criteria [9], and we compared demographic features among these classes.

For the aim of this study, we first calculated the number of median nerves showing prolonged DML (≥ 50% above ULN according to EFNS/PNS criteria) associated with SNCV slowing or absent SAP (e.g. consistent with CTS). Hence, we evaluated if the inclusion of prolonged DML changed significantly the proportion of electrophysiological diagnostic class of CIDP (from possible to definite, from not fulfilling to possible or definite) with multiple tests of proportions.

Afterward, we tested whether the median nerve might exhibit more severe demyelination at the carpal tunnel compared to the ulnar nerve at the wrist or and the same median nerve at the forearm. To do this, we compared the proportion of prolonged DML (≥ 50% above ULN) of median nerves (independently from the presence of sensory nerve conduction abnormalities) with the proportion of prolonged DML (≥ 50% above ULN) of ulnar nerves using a Chi-square test. As well, we compared both for median and ulnar nerves the proportion of electrophysiological abnormalities fulfilling EFNS/PNS criteria observed distally with the proportion of those found more proximally [i.e. motor nerve conduction velocity (MNCV) ≥ 30% below the lower limit of normal (LLN) along the forearm] using Chi-square test. Moreover, using regression analysis, we evaluated the relationship between DML of median and ulnar nerves and between DML and MNCV of both median and ulnar nerves. Lastly, we evaluated if the proportion of prolonged DML varied between diagnostic classes and if there were differences in temporal dispersion. STATA 13 was used for statistical analysis.

## Results

We analyzed data from 499 (out of 545) patients included consecutively into the Italian CIDP Database from January 2015 to January 2019. Forty-four patients were excluded from analysis due to the presence of a different cause for neuropathy, the unavailability of neurophysiological data, or the diagnosis (two patients) of chronic immune sensory polyradiculopathy (CISP) [10].

According to the EFNS/PNS criteria, 352 patients had a definite diagnosis of CIDP, 10 a probable diagnosis, 57 a possible diagnosis and 80 were not fulfilling the diagnostic criteria. There were no differences among diagnostic classes in age, gender and disease duration.

The total number of tested nerves (median and ulnar nerves) recorded in our database was 998, including motor conduction of 592 median nerves and 656 ulnar nerves, and sensory conduction of 521 median and 656 ulnar nerves.

Prolongation of DML fulfilling the EFNS/PNS criteria (≥ 50% above ULN) was found in 160 (27%) out of 592 median nerves. In 43 (27%) out of 160 median nerves sensory conduction was not performed, in 55/160 (34.3%) SNCV was reduced and in 53/160 (33.1%) SAP was absent.

Prolongation of DML without sensory conduction abnormalities was detected in only 9 (5.6%) median nerves. Patients with prolonged DML of median nerve had a median age of 56 years and a male to female ratio of 1.7:1 comparable to median age (56 years) and male to female ratio (1.8:1) of our CIDP population as a whole.

Prolongation of DML fulfilling the EFNS/PNS criteria (≥ 50% above ULN) was found in 141 (21.5%) out of 656 ulnar nerves. In 41 (29%) out of 141 ulnar nerves sensory conduction was not performed, in 44/141 (31.2%) SNCV was reduced and in 48/141 (34%) SAP was absent. Prolongation of DML without sensory conduction abnormalities was detected in only 8 (5.6%) ulnar nerves.

With the inclusion of the 108 median nerves that showed DML prolongation fulfilling the EFNS/PNS criteria and consistent with CTS for electrophysiological diagnosis of CIDP, 4 patients moved from possible to definite electrophysiological diagnostic class of CIDP and 2 patients moved from not fulfilling to possible diagnostic class; no patient moved from not fulfilling to definite diagnostic class. Statistical analysis proved that the inclusion of the 108 prolonged DML values did not change significantly the diagnostic electrophysiological class of CIDP (Table 1).

**Table 1:** Different frequencies of patient allocation in CIDP diagnostic classes considering and not considering electrophysiological criterion of prolonged DML (≥ 50% of upper limit of normal) of the median nerve consistent with CTS

	Standard EFNS/PNS criteria (%)	EFNS/PNS criteria with prolonged DML of the median nerve included (%)	Significance*
Definite	352/499 (70.6)	356/499 (71.4)	*p* = 0.83
Probable	10/499 (2)	10/499 (2)	
Possible	57/499 (11.4)	55/499 (11)	
Not fulfilling	80/499 (16)	78/499 (15.6)	

*CTS* carpal tunnel syndrome, *DML* distal motor latency

*Chi-square test for multiple proportion comparison

The statistical analysis evaluating whether median nerves might exhibit more severe demyelination at carpal tunnel showed that the proportion of DML prolongation was comparable between median and ulnar nerves (27% vs 21.5%; *p* = NS). Furthermore, MNCV reduction (≥ 30% below LLN) was found in 225 (38%) out of 592 median nerves and in 232 (35.4%) out of 656 ulnar nerves, disclosing a significantly higher proportion of demyelinating findings in the forearm than distal tract in both median (38% vs 27%; *p* = 0.02) and ulnar nerves (35.4% vs 21.5%, *p* < 0.001).

Linear regression analysis showed that DML in median nerve was directly related to DML in ulnar nerve and linear regression analysis showed that DML was inversely related to MNCV calculated in forearm both in median (*t*: − 8.09, *p* < 0.001) and ulnar (*t*: − 10.71; *p* < 0.001) nerves. We did not found any differences in the rate of prolonged DML nor temporal dispersion between diagnostic classes.

## Discussion

A primary concern of clinicians facing a suspected CIDP patient is to miss the diagnosis and to delay access to proper treatment. This led to develop several diagnostic criteria for CIDP [11]. EFNS/PNS criteria have the best combination of sensitivity and specificity but about one-fourth of patients may not satisfy electrodiagnostic criteria (EFNS/PNS sensitivity = 73%). Thus, clinical and supportive criteria play a major role in clinical practice in diagnosing CIDP when EFNS/PNS electrophysiological criteria are not satisfied [9].

Accordingly, we wondered if the inclusion of DML latency prolongation of median nerve could improve the diagnostic accuracy especially in the subset of patients not fulfilling the EFNS/PNS electrophysiological criteria.

We found that the inclusion of DML prolongation of median nerve does not improve significantly the diagnostic accuracy for CIDP. Indeed, DML inclusion overall changed diagnostic class in 6 (1.2%) out of 499 patients and electrodiagnostic class of CIDP changed from not fulfilling to possible in only 2 patients (2.5% of not-fulfilling patients).

Anyway, DML prolongation fulfilling EFNS/PNS criteria was quite common in our cohort of CIDP patients being present in about one-fourth of tested median nerves (27%).

Nevertheless, the statistical analysis did not reveal a higher proportion of abnormalities in the distal tract of median nerves rather than in ulnar nerves (27% vs 21.5%) as well distal slowing in median nerves was parallel to the distal slowing in ulnar nerves. To note, the male to female ratio of CIDP patients with median nerve electrophysiological findings consistent with CTS fully matched that of the entire cohort of CIDP patients, not supporting superimposed CTS.

Moreover, in median nerves DML prolongation was related to MNCV slowing, as well we did not observe a higher proportion of electrophysiological abnormalities fulfilling EFNS/PNS criteria at the wrist rather than to the forearm (i.e. prolonged DML vs reduced MNCV). These electrophysiological findings were similarly found in ulnar nerves hence pointing up a high concordance of demyelinating electrodiagnostic features between the upper limb nerves [12].

Taken together, our data suggest that demyelination that occurs distally in the median nerve generally follows a more widespread demyelinating process. This observation is in line with a previous description in literature, in which the comparison between CIDP and CTS patients showed a higher dCMAP duration and a higher duration/distal latency ratio in CIDP patients, a sign of broader demyelination [13]. Accordingly, our findings from a very large cohort of patients confirm that the median nerve at the carpal tunnel is not more prone to demyelination in line with previous reports in smaller samples of CIDP patients [6, 7].

In conclusion, we can infer that excluding DML prolongation of the median nerve does not increase the risk of missing a diagnosis of CIDP thus corroborating the current EFNS/PNS criteria.

## Data Availability

The data supporting the findings of this study are available on request from the corresponding author.
